# Individual Movement Strategies Revealed through Novel Clustering of Emergent Movement Patterns

**DOI:** 10.1038/srep44052

**Published:** 2017-03-08

**Authors:** Denis Valle, Sreten Cvetojevic, Ellen P. Robertson, Brian E. Reichert, Hartwig H. Hochmair, Robert J. Fletcher

**Affiliations:** 1 School of Forest Resources and Conservation, University of Florida, Gainesville, Florida, United States of America; 2 Department of Wildlife Ecology and Conservation, University of Florida, Gainesville, Florida, United States of America; 3 U.S. Geological Survey, Fort Collins Science Center, Fort Collins, CO, USA

## Abstract

Understanding movement is critical in several disciplines but analysis methods often neglect key information by adopting each location as sampling unit, rather than each individual. We introduce a novel statistical method that, by focusing on individuals, enables better identification of temporal dynamics of connectivity, traits of individuals that explain emergent movement patterns, and sites that play a critical role in connecting subpopulations. We apply this method to two examples that span movement networks that vary considerably in size and questions: movements of an endangered raptor, the snail kite (*Rostrhamus sociabilis plumbeus*), and human movement in Florida inferred from Twitter. For snail kites, our method reveals substantial differences in movement strategies for different bird cohorts and temporal changes in connectivity driven by the invasion of an exotic food resource, illustrating the challenge of identifying critical connectivity sites for conservation in the presence of global change. For human movement, our method is able to reliably determine the origin of Florida visitors and identify distinct movement patterns within Florida for visitors from different places, providing near real-time information on the spatial and temporal patterns of tourists. These results emphasize the need to integrate individual variation to generate new insights when modeling movement data.

Movement lies at the heart of several important problems. For instance, there has been considerable interest in analyzing human movement data to improve our understanding of human behavior^1^, of how people respond to disasters^2^, to determine the potential for disease spread or elimination^3^,^4^,^5^, and to aid urban planning^6^. Similarly, movement also plays key roles in ecology and conservation, enabling researchers to gain insight on animal behavior and helping to inform conservation planning such as the identification of important habitat for connectivity under climate change, the assessment of meta-population viability, and determining factors that influence spread of invasive species^7^,^8^.

Extracting information from movement data, however, is challenging. Various methods have been created to extract information from, and aid the visualization of the spatio-temporal patterns in, movement data^5^,^9^,^10^,^11^. Network methods, in particular, have been increasingly used in several disciplines^8^,^11^,^12^. This is possible because locations (e.g., towers for cell-phone data for human movement and habitat patches for animal movement) can be represented as nodes (or vertices) and movements between locations as links (or edges/arcs)^8^,^13^. As a result, several network metrics have been increasingly employed to summarize and interpret movement patterns^3^,^4^,^7^,^8^,^12^,^14^,^15^,^16^. Network analysis algorithms have also been devised to identify groups of users (often called communities or modules), such that these users have more connections (or stronger connections) within their group than expected by chance^12^,^17^,^18^,^19^. This is a useful task because it allows for the simplification and discovery of the underlying structure of these complex systems^20^. While these methods have traditionally clustered people without an explicit spatial focus, they have more recently been applied to the clustering of locations, yielding groups of locations such that there is more movement within than between groups^4^,^14^,^21^,^22^,^23^.

Here, we introduce a novel method for movement data that retains the original idea of using individuals as the clustering unit rather than locations. There are several benefits that arise when clustering individuals based on the locations they visit instead of clustering locations based on their connectivity to other locations. First, this approach allows for locations to have either single membership (a location is only used by a single group) or mixed membership (e.g., a location is used by multiple groups). As a result, it enables the identification of locations that are critical for the connectivity of the population (i.e., critical connectivity sites). These critical connectivity sites are vital in several fields, such as conservation biology^8^ and public health^5^. Second, grouping individuals rather than locations enables a more sensitive analysis of how individuals alter their behavior in the presence of environmental change. Third, clustering of individuals based on the locations they visit enables the assessment of how individual-level characteristics influence group membership. For instance, perhaps people from different nationalities or professions segregate into groups with different movement patterns. Similarly, wild animals might segregate into distinct movement groups due to differences in previous experiences (e.g., natal habitat effects) or due to despotic behaviors among individuals on the basis of their phenotypes^24^. Finally, movement data are notoriously hard to visualize^5^,^11^,^25^ and a typical approach is to summarize these data by calculating the amount of movement for each pairwise combination of locations^5^,^8^,^12^,^22^,^23^,^25^. By contrast, our method simplifies the visualization of movement patterns in a fundamentally different way by enabling users to examine movement patterns for different communities of individuals, which may yield unique insights.

We first present our statistical method and compare its performance against some commonly employed network analysis methods using simulated data. Next, we illustrate the unique insights that can be garnered by our method and its versatility with two case studies that vary dramatically both in terms of the amount of data and the size of the network. The first focuses on mark-recapture data from an endangered raptor, the snail kite (*Rostrhamus sociabilis*). This bird species has become a key indicator species for the Everglades’ restoration and, as a consequence, there is considerable interest in understanding how the landscape is used by this species and in identifying which sites are important for long-term connectivity^26^. The second case study examines movement patterns for Florida visitors using spatially explicit Twitter data. The tourism industry is a major industry in Florida and near real-time insights regarding Florida visitors (e.g., where they come from and where they visit within Florida) is important to improve planning of tourism-related activities and investments (e.g., tourism marketing).

## Results

### Simulations

**Figure 1:**
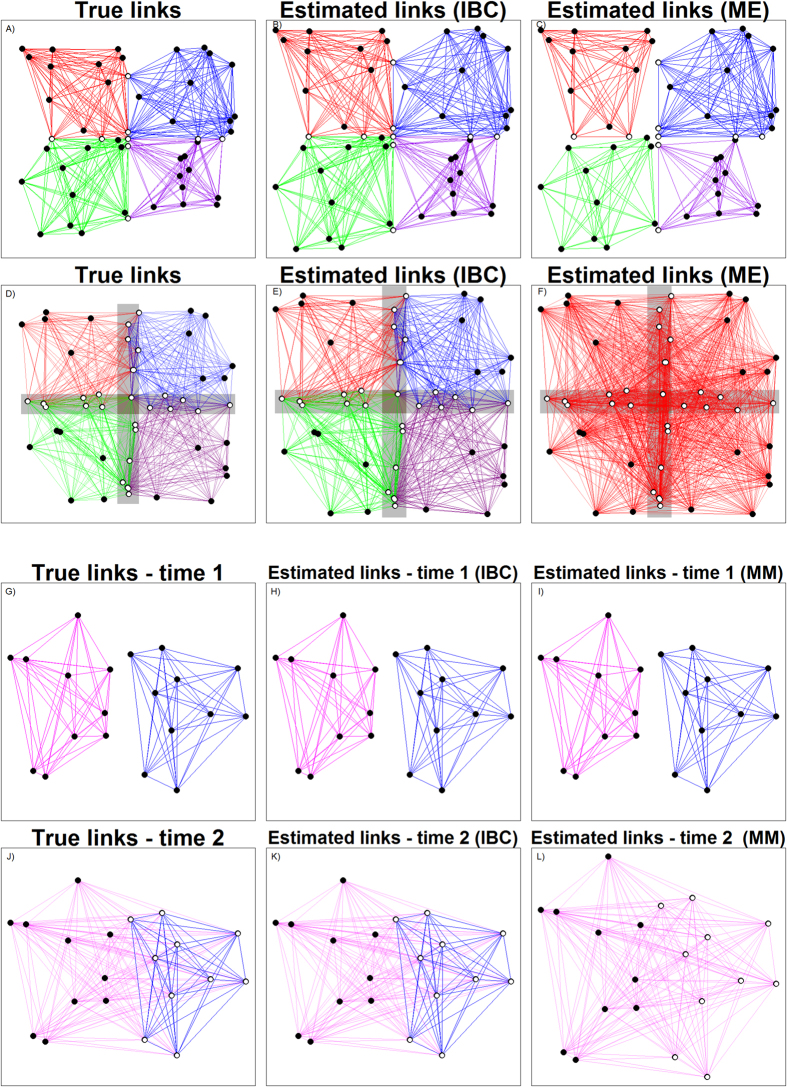
Simulated networks (left panels) and inference provided by different methods (middle and right panels). Left and middle panels (panels A, B, D, E, G, H, J and K) depict the simulated links and the links estimated by IBC, respectively, between sites assuming a 0.02 cuttoff. Right panels depict groups of locations estimated by the map equation method (ME; panels C and F) and modularity maximization (MM, panels I and L). Solid circles denote locations visited by a single group while hollow circles are locations visited by multiple groups. Panels A-F: simulated networks and inference for scenarios with different amount of mixed membership sites. Increased mixed membership is emphasized in panels D-F with grey polygons. The MM and latent cluster (LC) algorithms identified only 3 groups for scenario 1. For scenario 2, MM and LC algorithms identified 3 and 1 group, respectively (results not shown). Panels G-L: simulated networks and inference for scenarios where movement patterns change through time.

To illustrate the shortcomings of neglecting individual level information to identify mixed membership sites, we start by simulating data for two scenarios, one with few and the other with several mixed membership sites (Fig. 1A and D, respectively). We compare and contrast inference from our method (Individual-Based Clustering [IBC], fit using Markov-Chain Monte Carlo [MCMC] methods; see Methods) to that provided by network analysis methods commonly used for movement data. IBC was able to identify the correct number of groups (i.e., four) in both simulated examples. Furthermore, we were able to reliably estimate the underlying connectivity patterns between sites (Fig. 1B and E). On the other hand, the more traditional network algorithms used for movement data did not accurately capture the relationship between these locations. Even with a modest level of overlap (i.e., top panels in Fig. 1), the modularity maximization through simulated annealing (MM^7^,^27^) and latent cluster (LC^28^) algorithms failed to identify four groups. Only the map equation (ME^20^) algorithm successfully identified the four groups but, because this is a hard clustering method, it could only assign each site to a single group (Fig. 1C). With greater level of overlap (Fig. 1D), we find that all of the traditional methods used to cluster locations failed to identify the four existing groups, inferring instead the presence of only 1 (ME and LC; Fig. 1F) or 3 groups (MM).

Besides enabling the identification of mixed membership sites, our method can track how individuals change their movement patterns through time, which can yield insights unavailable from aggregate data. To illustrate this point, we generated data for two time periods, representing two groups (blue and purple) initially with limited connectivity (Fig. 1G). At the second time period, connectivity is increase through an invasion process, where the purple group invades the locations previously occupied solely by the blue group (Fig. 1J). IBC readily identifies these two groups and how their movement patterns have changed through time (Fig. 1H and K). On the other hand, to apply current network analysis methods to temporally dynamic data requires independent analysis on time slices of connectivity. Consequently, while these methods can generally detect the enhanced connectivity pattern in the second time period (Fig. 1L) relative to the first time period (Fig. 1I), these methods fail to identify which group was responsible for the invasion.

These results highlight how substantial information is lost when individual-level information is ignored. Furthermore, it is important to emphasize that our method also performs well when there is little overlap between groups (Fig. 1H). As a result, the proposed methodology is likely to be applicable to a wide range of problems, regardless of whether the underlying groups/communities do overlap on certain locations or not. Additional simulations where we vary the proportion of mixed membership sites, the number of communities, and the size of the network, reveal that our method tends to out-perform current network analysis methods (Appendix).

### Case study 1: landscape use by snail kites

**Figure 2:**
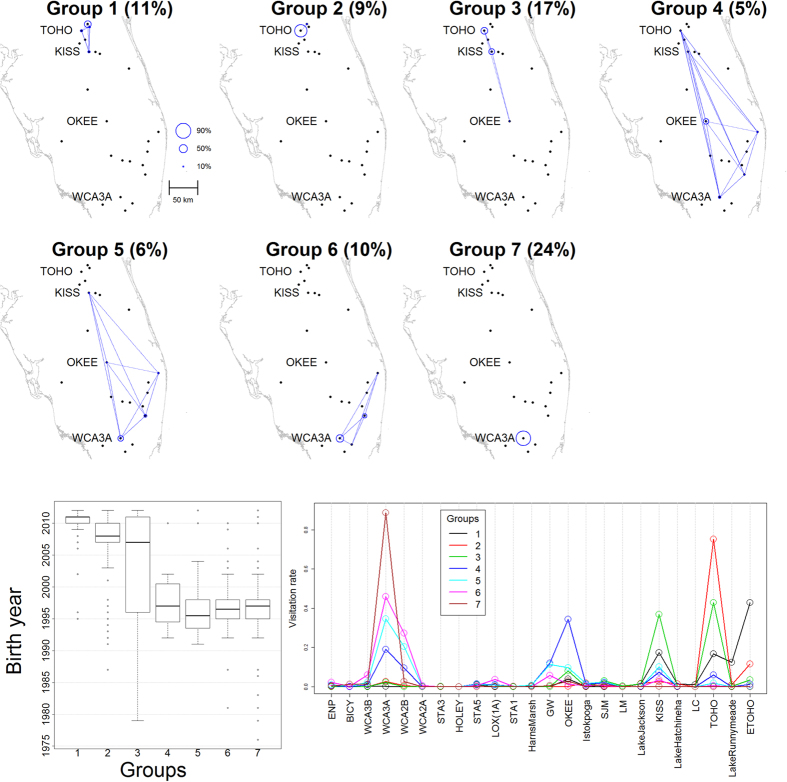
Individual variation in movement strategies by snail kites illustrate that these strategies vary by birth year and that some individuals show high site fidelity while others move throughout the geographic range. Maps: Numbers between parentheses refer to the proportion of all individuals in each group. Inserted text refers to the name of the main critical connectivity sites, where TOHO, KISS, OKEE, and WCA3A stand for Lake Tohopekaliga, Lake Kissimmee, Lake Okeechobee, and Water Conservation Area 3A, respectively. The size of blue circles is proportional to visitation rate *ψ*_*kl*_ and dashed lines connect all sites for which *ψ*_*kl*_ > 0.05. Boxplots: this plot shows the distribution of year of birth for individuals in each group. Bottom right graph: Visitation rate for different sites for the 7 groups (different colored lines). Sites are ordered from south to north (left to right). Maps were created in R^44^ (version 3.3.1) and the base map comes from the freely available GADM database^50^.

For our first analysis using data from all years, our algorithm identified 7 main groups encompassing 83% of all individuals. Thus, we focus our analysis on these 7 groups. Results from the algorithm reveal a clear north to south pattern of habitat use by the different groups (Fig. 2). Interestingly, there are striking differences in year of birth of individuals in these different groups despite the fact that this information was not part of the data used to fit this model. For instance, the northern groups (i.e., groups 1 and 2) are predominantly (96% and 85%, respectively) composed of individuals born after 2005 while the southern groups (i.e., groups 5, 6, and 7) are predominantly (94%, 95%, and 97%, respectively) composed of individuals born before 2005. These results suggest that landscape use is being stratified north to south by different bird cohorts, which reflects a known shift in the spatial distribution of reproductive output and juvenile recruitment over time, likely driven by increasing resource availability in the north^29^,^30^. These results also illustrate large variation in how individuals use the landscape, with some groups showing very high fidelity and few movements (where groups are confined to only a small number of wetlands) (e.g., groups 2 and 7), while other groups move throughout the entire system (e.g., groups 4 and 5). Movement across the entire system is particularly striking given the distances involved. For instance, group 4 utilizes both Lake Tohopekaliga (TOHO) and Water Conservation Areas 3A (WCA3A), two wetlands that are approximately 266 km apart. Finally, the bottom right graph in Fig. 2 suggests that Lake Okeechobee (OKEE), Lake Kissimmee (KISS), Lake Tohopekaliga (TOHO), and Water Conservation Areas 3A (WCA3A) are critical connectivity sites.

**Figure 3:**
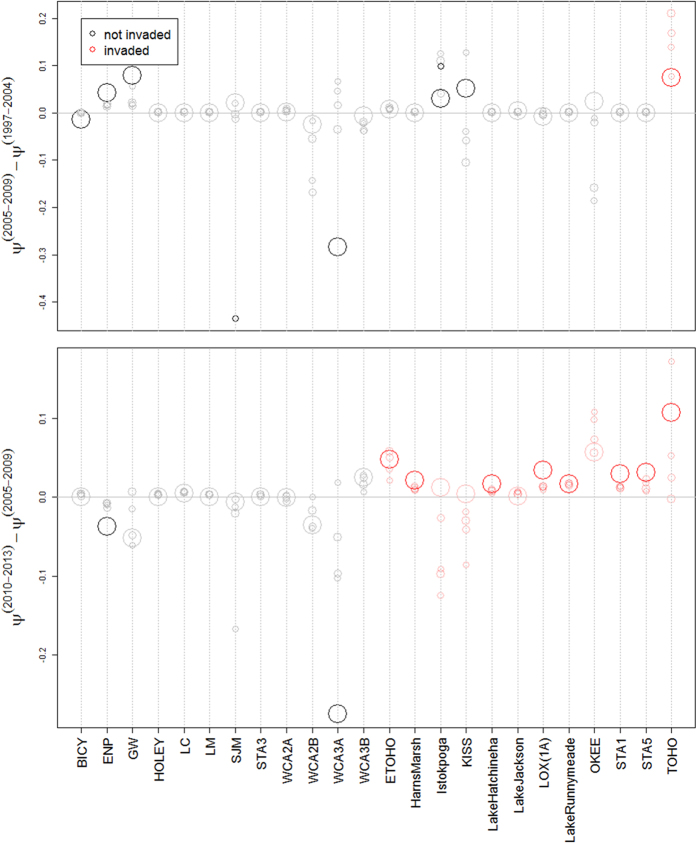
Changes in landscape use through time with increased prey availability from an exotic snail invasion. Panels depict changes in visitation rate for the five groups that jointly represent 97% of all individuals. The y-axis displays the difference of visitation rate for each site between periods *t*_1_ and *t*_2_, given by 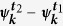. Circle sizes scale with the number of individuals in each group. Significant differences between time periods are shown in black (non-invaded sites) and red (invaded sites) while non-significant differences are shown in grey (non-invaded sites) and pink (invaded sites). Differences for which the 95% credible interval did not include 0 were deemed statistically significant.

One potential explanation for the differences in landscape use by these different bird cohorts is the enhanced resources provided by an exotic apple snail (*Pomacea maculata*) that invaded many of the northern habitat wetlands^29^. This provides us with a unique opportunity to assess how increased resources associated with the exotic snail invasion have changed landscape use through a more detailed analysis. Therefore, for our second analysis, we only include individuals born prior to 2005. Our algorithm identified 5 major groups, encompassing 97% of all individuals. Focusing on these 5 groups, our results reveal considerable changes in movement patterns of individuals with the exotic snail invasion (Fig. 3). When comparing the frequency with which different groups visited each site (i.e., visitation rate) before the exotic snail invasion (1997–2004) to the next time period when the only invaded site was TOHO (2005–2009), we find a substantial increase for TOHO and a significant decline for WCA3A. This is particularly noteworthy because WCA3A is one of our southernmost sites while TOHO is one of the northernmost sites, revealing a substantial geographic shift in how the landscape is used by these individuals. When comparing the period in which the only invaded site was TOHO (2005–2009) to the subsequent period in which multiple northern sites had been invaded (2010–2013), again we tend to find an increased visitation rate for the newly invaded sites, relative to those that were never invaded. Although these differences were statistically different from zero only for the most abundant group (which is comprised of 59% of the individuals born prior to 2005), taken together these results reveal how even relatively old individuals significantly changed their movement patterns to better exploit these enhanced resources.

### Case study 2: Twitter data

**Figure 4:**
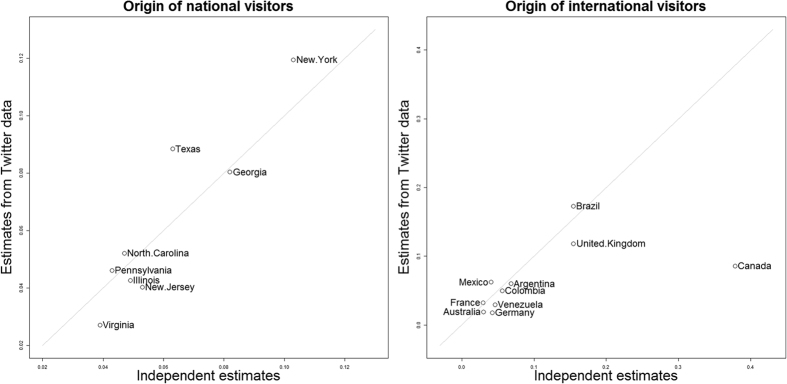
High correspondence between our estimates and independent estimates of state of origin of national visitors (left panel) and country of origin of international visitors (right panel). Independent estimates of visitation rate were extracted from ref. 31. The estimated visitation rate is based on summer data but is identical when using winter data. A 1:1 line was added for reference (diagonal grey line).

We start by examining movement patterns from all individuals that tweeted at least once from Florida and at least once during the summer and the winter. Based on the common assumption that people tweet the most at their place of residence^14^,^23^, we estimated that out of the 160,000 individuals in our dataset 48% were national visitors, 35% were Floridians, and 17% were international visitors. A separate analysis for national and international visitors revealed that the estimated origin of these visitors based on our method (Individual Based Clustering – IBC) closely matched independent estimates from Florida’s tourism marketing corporation^31^ (Fig. 4). For instance, after excluding Canada, we find a strong correlation (*r*_*pearson*_ = 0.932) between IBC’s and independent estimates of the proportion of individuals that came from each country for the top ten countries of origin. We also find a strong correlation (*r*_*pearson*_ = 0.928) when we do a similar analysis regarding the top states of origin for national visitors. We also find that all national and international visitor groups tended to visit Florida predominantly during the winter instead of the summer, except for visitors from the United Kingdom (UK) (results not shown).

**Figure 5:**
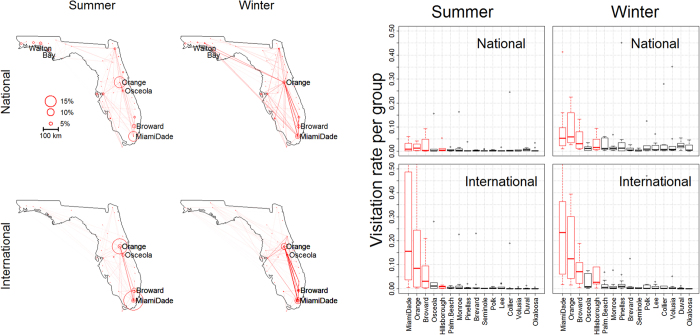
Differences in movement patterns for national and international Florida visitors. Left panels: National visitors travel more within Florida than international visitors and both types of visitors travel more within Florida during the winter than during the summer. Results from all 25 groups are presented simultaneously. Circle size for location l is proportional to the probability of visiting this location and belonging to group k (i.e., 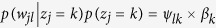). Darker lines indicate links from groups with more individuals. Right panels: Visitation rate for the 10 largest groups (y-axis) is shown for a subset of Florida counties. Only counties that were visited 2% of the times by more than one group are shown. Red boxes highlight the counties with the four largest international airports in Florida, as determined by passenger boarding in 2013^51^. Maps were created in R^44^ (version 3.3.1) and base map comes from the freely available database in the “maps” package in R (version 3.1.1).

By focusing on individuals rather than locations, our method provides an unprecedented level of information on these individuals regarding not only where visitors come from but also which places they choose to visit and at what time periods, extending previous research findings on global travel patterns extracted from tweets^14^. We find that international visitors tend to concentrate in and around Orlando and Miami, traveling little when compared to national visitors (maps in Fig. 5). Furthermore, both groups of visitors tend to travel more during the winter than during the summer. We also find that the majority of international visitor groups predominantly visit Orange, Broward, and Miami-Dade counties (right panel in Fig. 5). While this pattern may seem obvious in hindsight, given that these counties harbor large international airports (e.g., Broward, Orange, and Miami-Dade have Fort Lauderdale, Orlando, and Miami international airports, respectively), other counties with large international airports such as Tampa International Airport (i.e., Hillsborough county) are not as critical for connectivity as expected because few groups visit this county with high frequency. Furthermore, this extreme spatial concentration in visitation rate for international visitors becomes particularly evident when we compare these results to those from national visitors.

**Figure 6:**
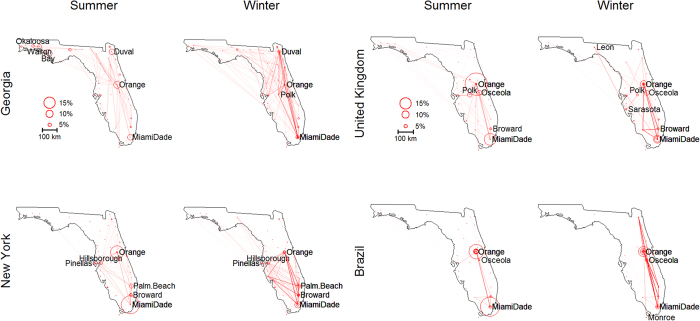
Differences in movement patterns according to state or country of origin of Florida visitors. Left panels: visitors from Georgia travel more, with greater visitation rate of northern Florida counties, than visitors from New York. Right panels: visitors from the United Kingdom (UK) travel more within Florida than Brazilian visitors. Results from all 25 groups are presented simultaneously. Circle size for location l is proportional to the probability of visiting this location and belonging to group k (i.e., 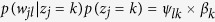). Darker lines indicate links from groups with more individuals. Maps were created in R^44^ (version 3.3.1) and base map comes from the freely available database in the “maps” package in R (version 3.1.1).

Differences in movement patterns are evident even within national visitors and international visitors. To illustrate this, we compare movement patterns of visitors from Georgia to those from New York. This comparison reveals that visitors from Georgia tend to travel much more throughout Florida, with a higher visitation rate of northern Florida counties, including Duval and counties in the panhandle (i.e., Okaloosa, Walton, and Bay). On the other hand, New Yorkers tend to travel mostly to Orange and southern counties (left panels in Fig. 6). We speculate that this difference arises because of mode of transportation. New Yorkers are likely to travel to Florida predominantly by plane while visitors from Georgia use more ground transportation and therefore spend more time in locations closer to Georgia, either as their final destination or en route to more southern destinations. Similarly, we can also examine how movement patterns differ for visitors of different nationalities (e.g., Brazil and United Kingdom). Our results suggest that Brazilian visitors tend to restrict themselves to Orlando and Miami while UK visitors travel more within Florida, including to counties rarely visited by Brazilians, such as Polk and Broward (right panels in Fig. 6). These differences in movement patterns may be associated with differences in the purpose of the trip (e.g., amusement parks and night club/dancing are activities reported at a much higher rate by Brazilian than UK visitors)^32^,^33^.

## Discussion

We have introduced a novel clustering algorithm for movement data that groups individuals with similar movement patterns. This perspective is a radical departure from current network analysis methods applied to movement data because it targets individuals rather than locations. There are several benefits of this approach. First, our method enables a more sensitive evaluation of changes in movement patterns potentially induced by a large-scale phenomenon (e.g., the exotic snail invasion) or seasonality (e.g., differences in visitation rates during the summer versus the winter). Second, our method allowed us to gain insight on how individual-level characteristics influence group membership and its movement patterns. In the case of the snail kite, it revealed how landscape use was stratified by different bird cohorts. For the Twitter data, our method revealed strikingly different movement patterns according to the state (or country) of origin of these visitors, which might be important for the strategic targeting of tourism marketing campaigns and investment planning (e.g., targeting investments to locations that attract higher spending tourists).

Third, we identified mixed-membership sites, something that is not possible in commonly used network analysis methods. For snail kites, we found four wetlands that were extensively used by multiple groups (OKEE, KISS, TOHO, and WCA3A). Similar results were obtained by Reichert *et al*.^26^ using network-based metrics with *a priori* identified groups based on age class and sex. However, we also found substantial temporal changes in how the different groups were utilizing these sites (Fig. 3). For instance, between 1997 and 2004, the main sites visited by multiple groups were clearly Water Conservation Area 3A and 2B (WCA3A and WCA2B, respectively), located in the South, suggesting that these sites should be prioritized for conservation. However, with time there was an increase in usage by multiple groups of several northern sites, particularly Lake Tohopekaliga (TOHO) and East Lake Tohopekaliga (ETOHO). These results highlight the challenge of determining priority conservation areas for highly mobile populations, particularly in the presence of changing environmental conditions (e.g., changes in food resource availability). In relation to human movement, our algorithm readily identified Broward, Orange, and Miami-Dade counties as important connectivity sites (right panel in Fig. 5). However, differences in visitation rate between sites were particularly extreme for international visitors, probably due to the restricted mobility of this group of people. One potential implication of these findings is that government investments geared towards increasing international tourism to Florida (due to substantially higher per capita spending of international visitors when compared to domestic visitors^34^) may, depending on the investment, generate economic impacts that are much more geographically concentrated than if these investments were focused on increasing national tourism. Finally, our method aids visualization by simplifying movement data through the grouping of individuals with similar movement patterns (e.g., Fig. 2), a fundamentally different approach than the use of pairwise summaries of the data often adopted by current methods^5^,^8^,^25^, enabling insights that would not be available otherwise (e.g., panels G-L in Figs 1 and 3).

More recently, other algorithms have been created that enable overlap between communities (e.g., refs 22,35, 36, 37). However, to our knowledge, these newer algorithms have not yet been employed to analyze movement data. Furthermore, these approaches do not share the other advantages of our method, which is partly due to the fact that these alternative approaches ignore individual-level information. For instance, by only taking into account the aggregate amount of movement between each pair of locations, these methods cannot provide insights into individual level determinants of group membership (e.g., year of birth). Another important feature of our method is that it does not require *a priori* specification of the number of groups. Instead, model users only pre-specify the maximum number of groups and the model determines if a smaller number of groups is warranted. Finally, our method appropriately represents the uncertainty in group membership and model parameters, a feature that several network analysis algorithms lack. Quantifying this uncertainty is critical to identify statistically significant changes in movement patterns (e.g., Fig. 3).

Some might argue that, given individual-level data, it is possible to retrieve essentially the same movement patterns we found by using carefully crafted queries instead of our method. For instance, Hawelka *et al*.^14^ used these queries to identify country of origin and destination of Twitter users. Our method differs from this approach in that it allows for the emergence of the underlying structure of the data while these queries require very specific hypotheses to be formulated before-hand. An important problem with our methodology is that it does not take into account imperfect detection. Ecologists have long acknowledged that the ability to detect individuals is likely to be influenced by habitat characteristics, which can alter conclusions for a variety of problems in ecology and conservation. This discussion is also relevant for Twitter data because different counties, states, and countries are likely to have different Twitter penetration rates. For instance, 28% of the original locations accounted for 99.8% of the tweets, suggesting that, in some regions of the world, Twitter data may fail to capture important movement patterns. Another problem is that, similar to many network approaches^8^, we evaluated the effect of large-scale phenomenon and individual-level characteristics on movement patterns by performing our analysis for different subsets of the data (e.g., snail-kite data restricted to different time periods or Twitter data restricted only to UK visitors). To conduct more formal inference on how individual-level characteristics influence group membership and the drivers of changes in movement patterns, one option would be to perform a two-step analysis, where we first obtain parameter estimates by running our model and then assess the determinants of these parameters using regression models. However, a more coherent Bayesian analysis would have these regressions embedded within our model, enabling the simultaneous estimation of all parameters. While multistate models^38^ may be an alternative modeling framework to perform this formal inference, one important drawback of these models is that they are heavily parameterized, often requiring users to arbitrarily aggregate sites into a handful of regions^39^,^40^. Furthermore, these multistate models have not been developed to capture the emergent movement patterns that are shown here.

Our method enables important insights. Our case study on snail kites, for example, reveals groups of site-faithful birds and groups of birds that move widely among wetlands, illustrating different movement strategies. Furthermore, we have found that recent cohorts of birds tend to rely much more on the northern lakes than older cohorts. This pattern can be explained by the recent invasion of exotic snails in the northern lakes leading to greater reproductive success^29^ coupled with the fact that snail kites tend to show breeding site fidelity (such that older cohorts may be less likely to move north to breed). Nevertheless, a more detailed analysis of the birds born prior to 2005 revealed there was a substantial change in movement patterns towards northern lakes after 2005. These findings illustrate the behavioral plasticity of snail kites in response to enhanced resources (i.e., due to the exotic snail invasion) as well as the difficulty of determining critical conservation sites in the presence of global change. Similarly, using Twitter data, our method revealed substantial differences in human movement patterns depending on state or country of origin of Florida visitors. These differences can potentially be attributed to differences in mode of transportation (Georgia visitors traveling more by car versus New Yorkers traveling predominantly by plane) and purpose of travel. Given the importance of the tourism industry to Florida, understanding who comes to Florida and their (often seasonal) movement patterns within Florida may be critical for the creation of more targeted tourism campaigns and to guide tourism investment decision-making in the State.

The striking correspondence between our estimates of the proportion of visitors from each state (or country) and independent data also highlight the large potential of obtaining detailed travel information (e.g., origin and visited locations at different spatial and time scales) in a faster and less expensive manner than using traditional methods such as surveys and airline company data, enabling near real-time analytics to aid tourism related decisions. However, there is admittedly an important discrepancy between our estimates and independent estimates of visitors from Canada. There might be several reasons for this discrepancy. For instance, Canadians may travel to Florida predominantly outside the period for which we collected Twitter data, Canadians may be comprised of an older group that is less likely to use Twitter, and/or our algorithm might be mistakenly assigning some of these individuals to the Floridian group if Canadians remain long periods of time in Florida. Additional work needs to be done to determine why there is such a large discrepancy between these estimates for Canadian visitors.

An area that merits future research refers to improving the way this model is fit. Although the speed of our MCMC scales linearly with the number of individuals and with the number of locations, this algorithm might still be too slow for extremely large datasets. Variational Bayes approaches^41^,^42^ might be better suited for these datasets, yielding faster (albeit approximate) results. Finally, combining Tweeter content and spatial location is likely to yield much richer insights regarding these groups of individuals than just location information. For instance, we hypothesize that an algorithm that uses content and spatial location information will be able to distinguish tourists from people traveling for business purposes. We believe that the method proposed here is an important addition to the tool-kit of scientists and businesses interested in movement patterns, enabling insights regarding site connectivity, the importance of individual sites to connectivity, group characteristics based on individual traits, and the impact of large-scale phenomena on movement patterns.

## Methods

### Description of statistical model

Our statistical model clusters individuals that have similar visitation rate for each location. We refer to it as Individual-Based Clustering (IBC). The generative model of our method is as follows. For each individual j, draw its group membership from


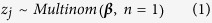


where *z*_*j*_ = *k* denotes that individual j belongs to group k. The vector ***β*** contains the probability that individuals come from each of these k=1,…, K groups. Given that individual j belongs to group k, then the number of times individual j is seen in locations 1,…, L ***w***_***j***_ = [*w*_*j*1 …_ *w*_*jL*_] is given by:


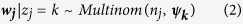


where *n*_*j*_ is the overall number of times individual j was seen. The vector ***ψ***_***k***_ contains the probability of this individual appearing in each location l given that this individual belongs to group k.

Borrowing ideas from nonparametric Bayesian models^43^, we adopt a truncated stick-breaking prior for the probability of individuals in group k (*β*_*k*_). In other words, we assumed that:


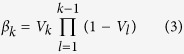


where *V*_*k*_~*Beta*(1, *α*) for k = 1,…, K − 1 and *V*_*K*_ = 1. We adopt this representation instead of the more standard Dirichlet prior distribution because it favors a small number of groups, circumventing the problem of *a priori* specifying the number of groups. Instead, we simply pre-define the maximum number of groups K. If the data supports a more parsimonious model, several groups will be empty or will have very few individuals, leading only to k* (k* < K) effective groups. Finally, we assume that the vector ***ψ***_***k***_ is drawn from:


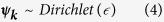


In all of our analyses, we set the maximum number of groups (K) to 25, our hyper-prior parameter *ε* to 0.1 (to make this prior uninformative) and *α* also to 0.1 (to promote fewer groups). Note that the maximum number of groups K is not constrained by the number of sites, differently from the standard network analysis methods that rely on locations as their sampling unit. To run this model, the basic input is a matrix with the number of times each individual (rows) is seen in each location (columns). Based on these assumptions and data, we fit this model in a Bayesian framework using the blocked Gibbs sampler similar to the one described in ref. 43. Full details regarding this Gibbs sampler can be found in the Appendix. We implemented this Gibbs sampler in C++ and R^44^,^45^,^46^ and provide the corresponding code in the Appendix.

It is important to note that our model does not directly model movement trajectory data; rather it uses information on the locations that individuals are observed to infer movement between these locations. While our approach can be easily modified to directly model movement data (in this case, ***w***_***j***_ would contain the number of times individual j performed each type of movement), this modification would significantly increase the number of parameters being estimated. For instance, in the current approach, the size of the vector ***ψ***_***k***_ is equal to the number of locations L. However, if we were modeling the amount of movement between each pairwise location while also allowing for individuals to remain in the same site, the size of the vector ***ψ***_***k***_ would be equal to 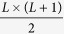 (if we ignore directionality) or *L*^2^ (if we account for directionality), where L is the number of locations. While these alternative model formulations can yield valuable insights, having a much larger number of parameters can substantially increase the challenge of visualizing and interpreting the output of the model. Furthermore, if data are limited, parameter estimates will have increasingly more uncertainty as the number of parameters increases. Finally, it is important to note that the data itself typically does not contain actual observations on movements. Rather, movement is often inferred by observing an individual in location A at time t and in location B at time *t* + Δ*t*. This censored nature of movement data is one of the main reasons that our model only focuses on where individuals were observed rather than their purported movement patterns.

### Simulations

To illustrate the potential limitations of network analysis methods commonly used for movement data in dealing with mixed membership sites (i.e., a site that is used by multiple groups of individuals) and changes in movement patterns through time, we compare and contrast inference from IBC to that provided by these other methods. First, we consider a model-based, latent network cluster model^28^. This model uses a Bayesian framework to identify clusters and requires the user to *a priori* specify the number of groups. This model is implemented in the package “latentnet” in R and we use a modified Bayesian Information Criterion (BIC) for selecting the number of groups. We also considered a modularity optimization algorithm that uses simulated annealing to identify the number and location of groups based on the approach described in Guimera and Amaral^27^ and Fletcher *et al*.^7^. This approach has been shown to be accurate in identifying groups or modules. Finally, we also considered the use of the ‘map equation’ algorithm, which identifies groups on networks based on the idea of information flow^20^. We implement this model using the “igraph” package in R. All these methods (latent network cluster model, modularity maximization, and the map equation) are hard clustering methods, where each node is assigned to only one group.

To compare these algorithms, we generated simulated data for multiple scenarios. In all scenarios, we considered 50 locations. For the first scenario (Fig. 1A), 41 locations are visited by just one group, and 9 locations are visited by two or more groups. In our second scenario (Fig. 1D), the setup is similar but the amount of overlap between groups is substantially higher (i.e., 27 locations out of 50 are visited by two or more groups). Finally, for our last scenario, we simulated 2 communities of individuals with initially very little connectivity (Fig. 1G) which then undergo dramatic changes in connectivity due to an invasion process (Fig. 1J). In this invasion process, one community (purple in Fig. 1) invades the locations previously utilized mainly by the second community (blue in Fig. 1). Using these simulated data, we fit IBC and compare its results to those from the latent cluster model (LC), modularity maximization via simulated annealing (MM), and the map equation (ME). Additional details regarding how these data were simulated can be found in the Appendix.

### Case study 1: landscape use by snail kites

Our first case study focuses on movement patterns of snail kites (*Rostrhamus sociabilis plumbeus*) in Florida, a bird species that has become a key indicator species for Everglades’ restoration. The snail kite is an endangered raptor that inhabits the freshwater wetlands in peninsular Florida^47^. This raptor is an extreme dietary specialist that used to feed almost exclusively on the native Florida apple snail (*Pomacea paludosa*)^47^. However, with the appearance of the exotic snail (*Pomacea maculata*) around 2005 on Lake Tohopekaliga (TOHO) and its subsequent spread throughout many of the northern wetlands, these exotic snails have become an important novel food resource for the snail kite population^29^,^48^.

Our data consisted of multiple standardized mark-recapture surveys conducted yearly from 1997 to 2013 during the peak of the snail kite breeding season (March 1–June 30). In these surveys, technicians attempt to locate all snail kite nests and band all nestlings found at these nests just prior to fledging, such that the age of nearly all banded birds is known^49^. Band-resight surveys span the entirety of the snail kite’s known breeding range in Florida, providing a long-term record of movement patterns of individuals across their breeding range.

We performed two analyses. For the first analysis, our goal was to quantify the general movement patterns of individual snail kites. To this end, we aggregated observations from 1,151 individuals into a single matrix that contained the number of years in which each individual was observed at each location (from a total of 24 wetlands). For the second analysis, we focused on how movement patterns changed through time, potentially due to changing resource availability. To do this, we retained just individuals born before 2005 (n = 725) and ran our analysis for the movement data prior to the exotic snail invasion (i.e., <2005). This analysis generated the parameters ***z***^**1997**–**2004**^, ***β***^**1997**–**2004**^ and ***ψ***_***k***_^**1997**–**2004**^, where the superscripts denotes the time interval of the dataset used to estimate these parameters. Then, to determine how movement patterns changed post-2005, we retained the group membership status of each individual (i.e., ***z***^**1997**–**2004**^) but estimated a new set of parameters ***ψ***_***k***_^**2005**–**2009**^ and 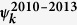. We chose these two time periods because only the TOHO site had been invaded by the exotic snails between 2005 and 2009 while from 2010 onwards many northern wetlands had exotic snails established^29^. A comparison of the vector ***ψ***_***k***_ for different time periods identifies how habitat use has changed for the same set of individuals before and at different times after the exotic snail invasion.

### Case study 2: movement patterns of Florida visitors based on Twitter data

We gathered worldwide Twitter data from summer (August-October 2014) and winter (December 2014–February 2015) through the Twitter streaming API and retained only those tweets that were geotagged (i.e., tweets with exact geographic coordinates). Because we were interested in movement patterns of Florida visitors and how these patterns changed with season, we only retained people that tweeted at least once in Florida during this time period and that tweeted at least once during the summer and the winter, regardless of region. These data were then summarized in one matrix per season (summer and winter) containing the number of tweets per person in each location. We relied primarily on the following spatial aggregations to define these locations: data from within Florida were aggregated at the county level (total of 67 counties), data within the US but outside Florida were aggregated at the state level (total of 50 states minus Florida plus the District of Columbia), and data from outside the US were aggregated at the country level. We eliminated locations with less than 1,000 tweets, resulting in a final dataset containing 98 locations (28% of the original locations) but virtually all tweets (99.8% of all the tweets). Our final dataset contained approximately 26 million tweets from 160,000 individuals. These data were then further spatially aggregated and subsets of individuals and locations extracted depending on the purpose of the different analyzes performed, as detailed below.

We performed our analysis in several steps. The first step consisted in identifying three types of individuals: individuals that predominantly tweet from Florida (i.e., assumed to be Floridians), within the US but outside Florida (i.e., assumed to be national visitors), and from abroad (i.e., assumed to be international visitors). For this analysis, we aggregated all locations within Florida into a single location (“Florida”) and all locations in the other US states as another location (“rest of US”). Our second step consisted of conducting separate analyses for each of these groups. To determine the state of origin of national visitors, we examined the movement patterns within the US of these visitors. To this end, we aggregated tweets from within the US by state and tweets from abroad into a single “abroad” location. To determine the country of origin of international visitors, we examined movement patterns across the world for these individuals. Thus, data were aggregated by country, “rest of US”, and Florida. In these analyses, the proportion of individuals from location l was estimated by *β*_*k*_, where k* is the group that visited location l the most. To further illustrate the detailed spatial and temporal insights that can be gained from these data using our method, our third step consisted of a closer examination of movement patterns within Florida of subsets of national and international visitors. For this purpose, we arbitrarily selected national visitors from Georgia and New York and international visitors from Brazil and the UK and compared and contrasted the movement patterns within Florida of these different groups of visitors and as a function of season using only tweets from within Florida.

To determine how movement patterns change for the same set of individuals from summer to winter, we adopt a methodology that is similar to the one we used for the snail kite. First, we fit our model to summer data, obtaining estimates for ***z***^***summer***^, ***β***^***summer***^ and 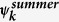. Then, we estimate 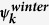 from the winter data while maintaining the group membership status the same.

## Additional Information

**How to cite this article**: Valle, D. *et al*. Individual Movement Strategies Revealed through Novel Clustering of Emergent Movement Patterns. *Sci. Rep.* **7**, 44052; doi: 10.1038/srep44052 (2017).

**Publisher's note:** Springer Nature remains neutral with regard to jurisdictional claims in published maps and institutional affiliations.

## Supplementary Material

Supplementary Information
