# Supplementary material for: Individual Movement Strategies Revealed through Novel Clustering of Emergent Movement Patterns
Source: Sci Rep. 2017 Mar 8;7:44052. doi: 10.1038/srep44052 (PMC5341027; doi:10.1038/srep44052)
Supplement: Supplementary Information [file srep44052-s1.pdf]

Supplementary information for:

“Individual Movement Strategies Revealed through Novel Clustering of Emergent Movement Patterns”.

Denis Valle[1]*, Sreten Cvetojevic[1], Ellen P. Robertson[2], Brian E. Reichert[2], Hartwig H. Hochmair[1], Robert J. Fletcher, Jr.[2]

[1] School of Forest Resources and Conservation, University of Florida, Gainesville, Florida, United States of America

[2] Department of Wildlife Ecology and Conservation, University of Florida, Gainesville, Florida, United States of America

* Corresponding author. Email: drvalle@ufl.edu.

<u>Derivation of the full conditional distributions employed for the blocked Gibbs sampler</u>

The complete model is given by:

$$p(\boldsymbol{z}, \boldsymbol{\Psi}, \boldsymbol{\beta} | \boldsymbol{W}, \boldsymbol{n}) \propto \left\{ \prod_{j=1}^{J} \prod_{k=1}^{K} p\left(\boldsymbol{w_j} \middle| n_j, z_j = k, \boldsymbol{\psi_k}\right)^{I(z_j=k)} \right\} \left\{ \prod_{j=1}^{J} p\left(z_j \middle| \boldsymbol{\beta}\right) \right\} \left\{ \prod_{k=1}^{K} p(\boldsymbol{\psi_k} | \epsilon) \right\} p(\boldsymbol{\beta} | \alpha)$$

where:

- $\boldsymbol{W}$ is a J x L matrix (J and L are the total number of individuals and locations, respectively) with rows containing the number of times individual j was seen in each location l=1,…,L ($\boldsymbol{w_j} = [w_{j1} \quad \cdots \quad w_{jL}]$);

- $\boldsymbol{n}$ is a vector of size J containing the overall number of times each individual j was seen (n_j);

- $\boldsymbol{\Psi}$ is a K x L matrix (K is the total number of groups) with rows containing the probability of observing individuals from group k in each location ($\boldsymbol{\psi_k} = [\psi_{k1} \quad \cdots \quad \psi_{kL}]$);

- $\boldsymbol{\beta}$ is a vector of size K containing the probability of belonging to each group; and

- $\boldsymbol{z}$ is a vector of size J containing the group membership status of each individual j (z_j); and

- $I()$ is an indicator function which takes the value of one if the condition within the parentheses is satisfied and zero otherwise.

This expression can be re-written more explicitly as:

$$\propto \left\{ \prod_{j=1}^{J} \prod_{k=1}^{K} Multinom\left(\boldsymbol{w_j} \middle| n_j, \boldsymbol{\psi_k}\right)^{I(z_j=k)} \right\} \left\{ \prod_{j=1}^{J} Multinom\left(z_j \middle| n = 1, \boldsymbol{\beta}\right) \right\}$$

$$\times \left\{ \prod_{k=1}^{K} Dirichlet\left(\boldsymbol{\psi_k} \middle| \epsilon\right) \right\} \left\{ \prod_{k=1}^{K-1} Beta(V_k | 1, \alpha) \right\}$$

where $V_K = 1$ and $\beta_k = V_k \prod_{i=1}^{k-1}(1 - V_i)$. Throughout our different analyses, we set the hyper-parameter ϵ to 0.1 to make it uninformative while the hyper-parameter α was also set to 0.1 to promote a more parsimonious model with fewer groups.

We fit this model within a Bayesian framework, using a Blocked Gibbs sampler similar to the one described in [1]. The full conditional distributions (FCDs) are given below:

- FCD for the probability of individuals in group k being seen at each location $\boldsymbol{\psi_k}$:

$$p(\boldsymbol{\psi_k}|\ldots) \propto \left[\prod_j Multinom\left(\boldsymbol{w_j}\middle|n_j, \boldsymbol{\psi_k}\right)^{I(z_j=k)}\right] Dirichlet(\boldsymbol{\psi_k}|\epsilon)$$

$$p(\boldsymbol{\psi_k}|\ldots) = Dirichlet([s_{k1} + \epsilon, \ldots, s_{kL} + \epsilon])$$

where s_{kl} is the number of times individuals from group k were seen in each location l (l=1,…,L).

- FCD for the latent group membership variable for individual j z_j:

$$p\left(z_j = k\middle|\ldots\right) = \frac{Multinom\left(\boldsymbol{w_j}\middle|n_j, \boldsymbol{\psi_k}\right) \times V_k \prod_{i=1}^{k-1}(1 - V_i)}{\sum_{r=1}^{R} Multinom\left(\boldsymbol{w_j}\middle|n_j, \boldsymbol{\psi_r}\right) \times V_r \prod_{i=1}^{r-1}(1 - V_i)}$$

We drew z_j from a multinomial distribution with size 1 and probabilities given by the expression above.

- FCD for the stick-breaking parameters V_k:

$$p(V_k|\ldots) \propto Binomial(q_k|n = q_k + q_{>k}, V_k) Beta(V_k|1, \alpha)$$

$$p(V_k|\ldots) = Beta(q_k + 1, q_{>k} + \alpha)$$

where q_k is the number of individuals assigned to group k and $q_{>k}$ is the sum of the number of individuals in groups k+1,…,K ($q_{>k} = q_{k+1} + \cdots + q_K$).

Code to fit the Blocked Gibbs Sampler

Simulated data

The data required by our method consist of a matrix with the number of times each location was visited by each individual. In this matrix, rows are individuals and columns are locations. For instance, if we had four locations and locations 1-4 were visited 2, 3, 1, and 1 times, respectively, our observation for individual j would consist of the following vector $w_j = [2, 3, 1, 1]$.

The data required for the network analysis methods consist of a matrix with the amount of movement between all pairwise combinations of locations. To generate this input, we randomly sample without replacement the locations visited by each individual to then determine the amount of movement between the different locations. For instance, using the example above, we start by creating a derived vector with the explicit identifiers of each location $r_j = [1, 1, 2, 2, 2, 3, 4]$. We then randomly sample these location identifiers without replacement. Say that this process generates the vector $r_j^* = [2, 2, 1, 1, 3, 2, 4]$, where now the elements in this vector represent the location visited at each time point. This vector can then be represented by the following matrix

$$R_j = \begin{bmatrix} 1 & 0 & 1 & 0 \\ 1 & 1 & 0 & 1 \\ 0 & 1 & 0 & 0 \\ 0 & 0 & 0 & 0 \end{bmatrix}$$

where numbers refer to the amount of movement departing from location i (row i) and arriving in location s (column s). The input matrix required for the network analysis methods is obtained by summing these matrices over all individuals j. To illustrate this process, we provide below the code we used to create the simulated data shown in panel D of Fig. 1 in the main manuscript.

```
rm(list=ls(all=TRUE))
set.seed(1)

#set up the initial parameter values
nloc=50 #number of locations
inds=list(ind1=c(1:12,19:24,50),ind2=c(13:24,31:36,50),
          ind3=c(25:36,43:50),  ind4=c(6:12,37:50)) #locations visited by each group
psi=numeric()
for (i in 1:4){
  tmp=runif(nloc,min=0.5,max=1.1)
  base1=rep(0.001,nloc)
  base1[inds[[i]]]=tmp[inds[[i]]]
  psi=rbind(psi,base1/sum(base1))
}

beta=c(0.2,0.3,0.3,0.2)
nind=10000 #number of individuals
nmov=rpois(nind,lambda=10) #number of times each individual is seen
psi=t(psi)

#generate the fake data
res=matrix(NA,nind,nrow(psi))
locloc=matrix(0,nloc,nloc)
resz=rep(NA,nind)
```

```r
for (i in 1:nind){
  #generate z's
  tmp=rmultinom(1,size=1,prob=beta) #sample group membership of each indiv
  z=which(tmp==1)
  resz[i]=z

  tmp=rmultinom(1,size=nmov[i],prob=psi[,z]) #sample where each indiv is seen
  res[i,]=tmp

  #summarize this information in a location-by-location table
  tmp1=numeric()
  for (j in 1:nloc) tmp1=c(tmp1,rep(j,tmp[j]))
  tmp2=sample(tmp1)
  for (j in 2:length(tmp2)){
    ind1=tmp2[j-1]
    ind2=tmp2[j]
    locloc[ind1,ind2]=locloc[ind1,ind2]+1
    locloc[ind2,ind1]=locloc[ind2,ind1]+1
  }
}

#output the results
write.csv(res,'sim1 indiv data.csv',row.names=F)
write.csv(locloc,'sim1 locloc data.csv',row.names=F)
write.csv(psi,'sim1 parameter.csv',row.names=F)
```

The outputs from this code are:

1. the individual level data "sim1 indiv data.csv" used by our method IBC;
2. the spatially aggregated data "sim1 locloc data.csv" used by common network analysis methods; and
3. the underlying visitation rate parameters for each group "sim1 parameter.csv".

The figure below shows the assumed visitation rate for each group, where dotted red lines indicate locations that are frequently visited by individuals from various groups.

```r
setwd('U:/independent studies/movement LDA/appendix code')
psi=read.csv('sim1 parameter.csv')
ind=c(19:24,31:36,43:50,6:12)
par(mfrow=c(4,1),mar=c(2,2,1,1),oma=c(3,3,0,0))
psi=t(psi)
for (i in 1:4) {
  plot(psi[i,],type='h',ylim=c(0,0.08))
  abline(v=ind,col='red',lty=2) #locations used by multiple groups
  text(0,0.07,paste('Group',i),pos=4,cex=2)
}
mtext(side=1,at=0.5,outer=T,line=1,'Locations',cex=2)
mtext(side=2,at=0.5,outer=T,line=1,'Visitation rate',cex=2)
```

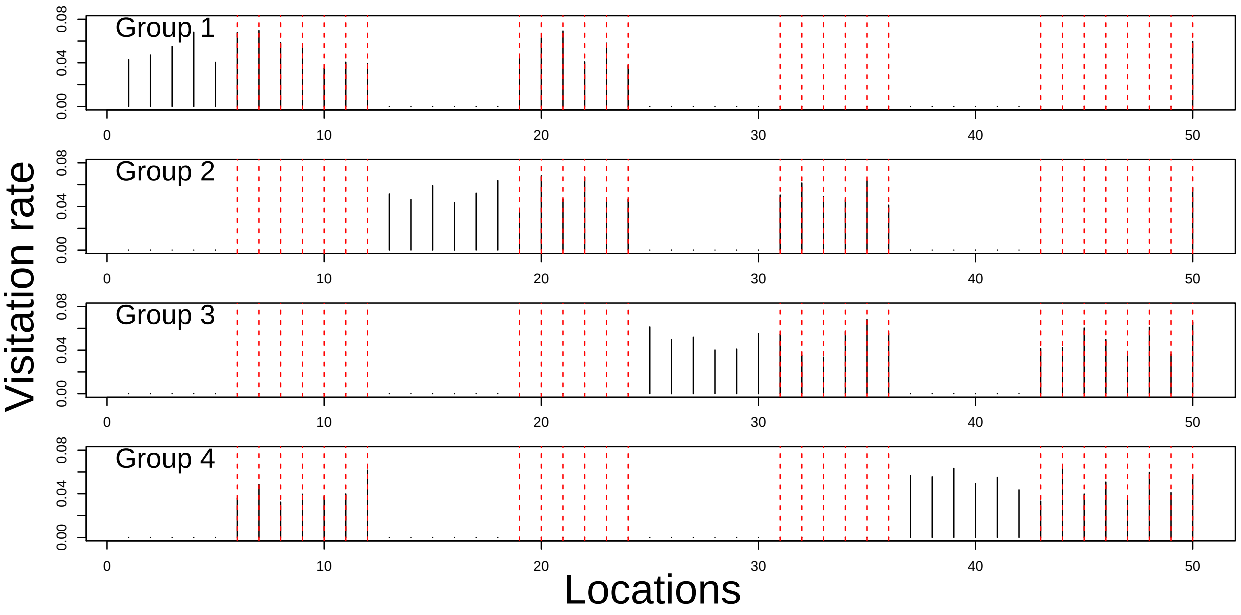

Blocked Gibbs sampler

Our algorithm is primarily implemented in R, with some auxiliary functions implemented in C++ through the use of the R package "Rcpp". To fit this model, we used the following code:

```
set.seed(10)
library(gtools)
library(Rcpp)

setwd('U:/independent studies/movement LDA/appendix code')
source('gibbs functions.R')
sourceCpp("gibbs_functions_cpp.cpp")

#import data (rows are individuals, columns are locations, cells contain the number
#of times each individual was seen at each location)
dat=data.matrix(read.csv('sim1 indiv data.csv',as.is=T))

ngroups=25 #maximum number of groups
alpha=0.1 #hyper prior parameter
epsilon=0.1 #hyper prior parameter
ngibbs=1000 #number of iterations for the Gibbs Sampler

#run Gibbs sampler
results=gibbs.sampler(ngroups,ngibbs,dat,alpha,epsilon)

write.csv(results$loglikel,'loglikel.csv',row.names=F)
write.csv(results$beta,'betas.csv',row.names=F)
write.csv(results$psi,'psi.csv',row.names=F)
```

This code imports the individual level data "sim1 indiv data.csv" and relies on the wrapper function "gibbs.sampler" (contained in the file "gibbs functions.R") to fit the model. The arguments of this function are:

1. ngroups: the maximum number of groups;
2. ngibbs: number of iterations for the Gibbs sampler;
3. dat: the data; and
4. alpha and epsilon: values for the hyper-prior parameters α and ϵ.

The output of this function consists in a list containing the following parameters and results for each Gibbs iteration: $\mathbf{z}$,$\mathbf{V}$,β,ψ and the log-likelihood (a useful metric to assess algorithm convergence).

To assess if the algorithm has converged, we examine the trace-plot of the log-likelihood. The code below suggests that the algorithm has converged in 100 iterations. It is important to note that our algorithm does not necessarily result in a model with the highest likelihood because our truncated stick-breaking prior will attempt to enforce a more parsimonius representation (i.e., a smaller number of groups).

```
loglikel=read.csv('loglikel.csv',as.is=T)
plot(loglikel$x,type='l',ylim=c(-298000,-296000),ylab='Log-likelihood',xlab='Iterations')
abline(v=100,col='grey')
```

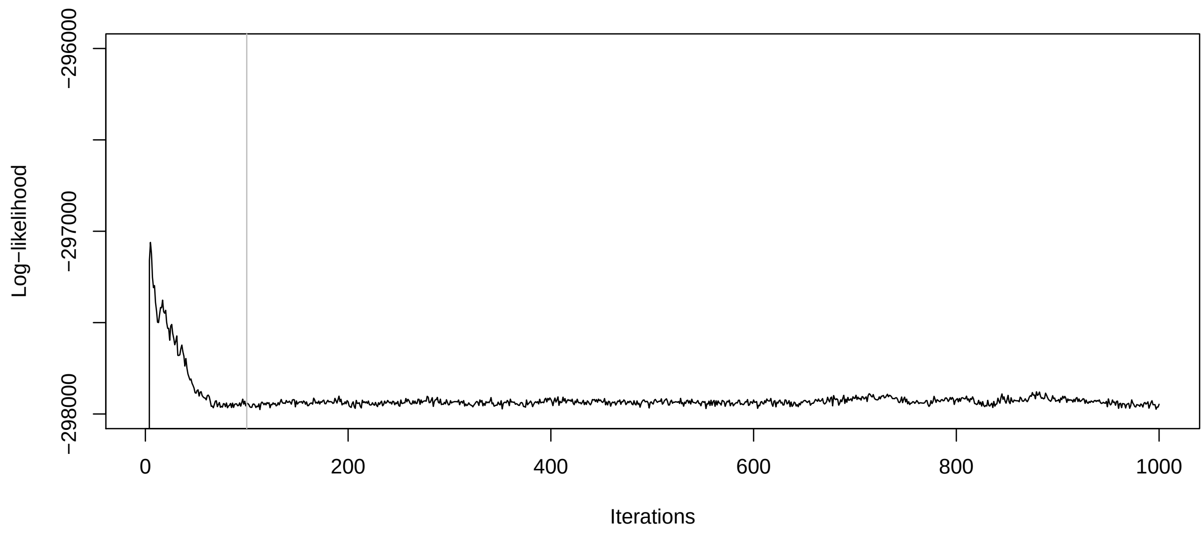

Next, we determine which are the main groups detected by the algorithm. The code below suggests that there are only 4 major groups: groups 2, 7, 10, and 11.

```
betas=read.csv('betas.csv',as.is=T)
seq1=100:nrow(betas)
betas1=apply(betas[seq1,],2,mean)
plot(betas1,type='h',xlab='Groups',ylab=expression(beta))
```

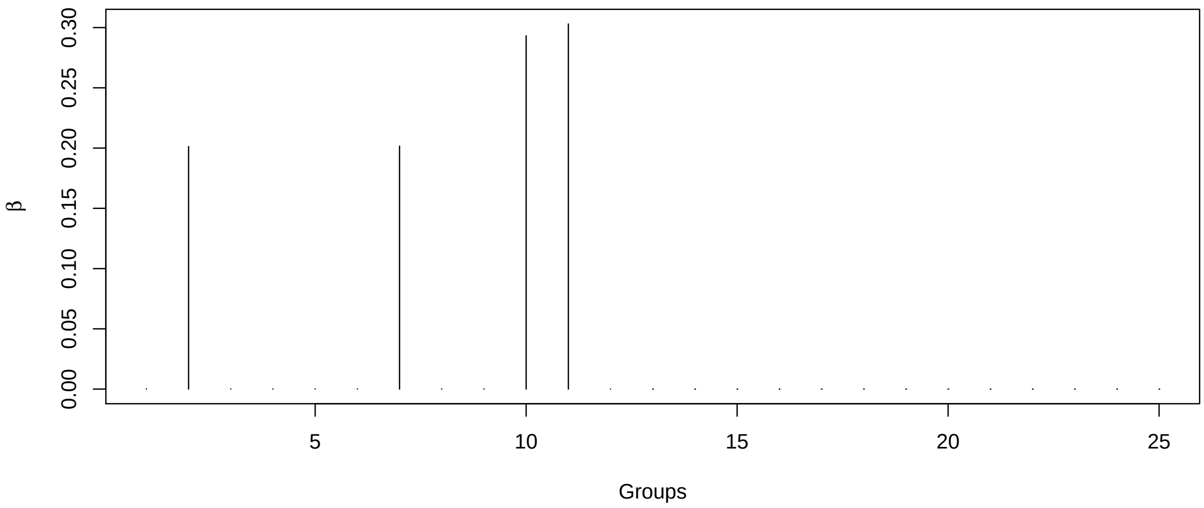

Finally, we compare the estimated visitation rate of each group with the true model parameters in "sim1 parameter.csv". This comparison reveals that our model is able to estimate the true visitation rate ψ parameters very well:

```
psi=data.matrix(read.csv('psi.csv',as.is=T))
seq1=100:nrow(psi)
psi1=apply(psi[seq1,],2,mean)
ngroups=25
nloc=50
psi2=matrix(psi1,ngroups,nloc)[c(2,7,10,11),]

#re-arrange groups to match true configuration of groups
psi3=psi2[c(2,3,4,1),]
# for (i in 1:4) plot(psi3[i,],type='h')

true=data.matrix(read.csv('sim1 parameter.csv',as.is=T))
par(mfrow=c(1,1),mar=c(6,6,1,1))
max1=max(c(true,psi3))
rango=c(0,max1)
plot(t(true),psi3,xlim=rango,ylim=rango,xlab='true parameters',ylab='parameter estimates',
     cex.axis=1.5,cex.lab=2,cex=2)
lines(rango,rango,col='grey')
```

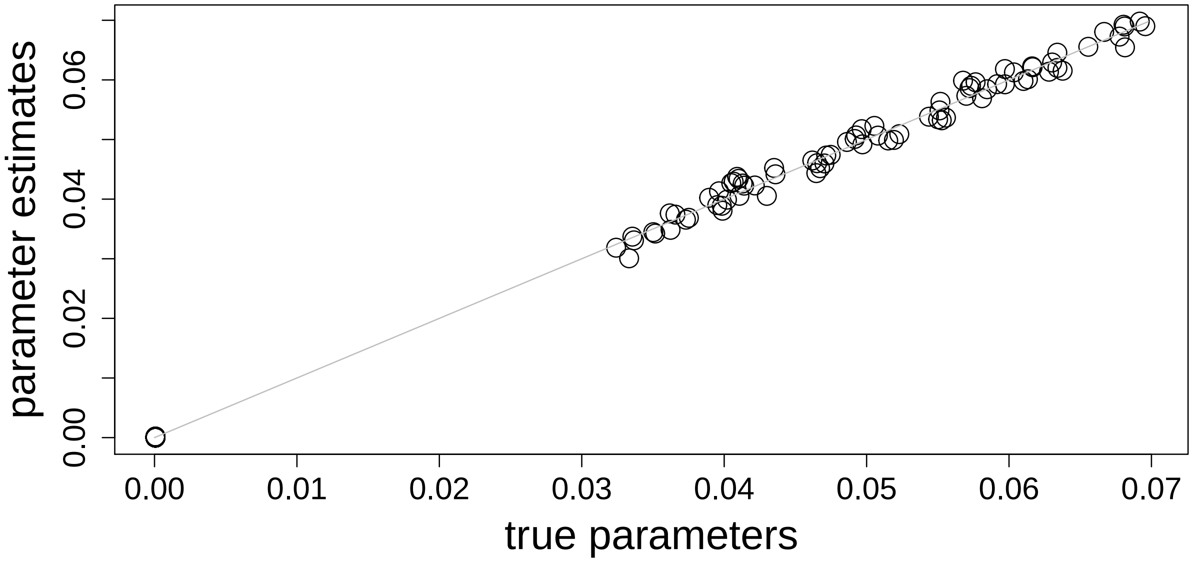

To fit our IBC model, we relied on two auxiliary files:

1. "gibbs functions.R": this file contains the wrapper function "gibbs.sampler", functions to sample from the full conditional distributions of each set of parameters ("update.z", "update.vh", "update.psi"), and a function to calculate the log-likelihood "calc.loglikel"; and
2. "gibbs_functions_cpp.cpp": this file contains functions written in C++ that speed up some of the calculations required in the "update.z" function.

Here is the code within the file "gibbs functions.R":

```
gibbs.sampler=function(ngroups,ngibbs,dat,alpha,epsilon){
  nind=nrow(dat)
  nloc=ncol(dat)
  nvisits=apply(dat,1,sum)

  vh=runif(ngroups-1)
  vh=c(vh,1)
  z=sample(1:ngroups,nind,replace=T)
  param=list(z=z,vh=vh,psi=matrix(1/nloc,ngroups,nloc,byrow=T))

  #store results
  vec.psi=matrix(NA,ngibbs,ngroups*nloc)
  vec.z=matrix(NA,ngibbs,nind)
  vec.vh=matrix(NA,ngibbs,ngroups)
  vec.beta=matrix(NA,ngibbs,ngroups)
  vec.loglikel=rep(NA,ngibbs)
  for (i in 1:ngibbs){
    print(i)
    tmp=update.z(param,nind,ngroups,nloc)
    param$z=tmp$z
    param$pi.h=tmp$pi.h
    param$vh=update.vh(param,ngroups,alpha,nind)
    param$psi=update.psi(param,ngroups,nloc,epsilon)
```

```
    vec.z[i,]=param$z
    vec.vh[i,]=param$vh
    vec.beta[i,]=exp(param$pi.h[1,])
    vec.psi[i,]=param$psi
    vec.loglikel[i]=calc.loglikel(param,ngroups,nloc)
  }

  list(z=vec.z,vh=vec.vh,beta=vec.beta,psi=vec.psi,loglikel=vec.loglikel)
}

update.z=function(paramz,nind,ngroups,nloc){

  #calculate probabilities
  pi.h=loglikel=matrix(NA,nind,ngroups)
  for (j in 1:ngroups){
    if (j==1) pi.h[,j]=log(paramz$vh[j])
    if (j!=1) pi.h[,j]=log(paramz$vh[j])+sum(log(1-paramz$vh[1:(j-1)]))
    loglikel[,j]=(dat*matrix(log(paramz$psi[j,]),nind,nloc,byrow=T))%*%rep(1,nloc)
  }

  res=loglikel+pi.h
  max0=getmax(res,nrow(res),ncol(res))
  max1=matrix(max0,nind,ngroups)
  res1=exp(res-max1)
  res2=res1/matrix(res1%*%rep(1,ngroups),nind,ngroups)

  #multinomial draw
  cumsum1=cumsummat(res2,nrow(res2),ncol(res2))
  res3=cbind(0,cumsum1)
  uni=runif(nind)
  z=rep(NA,nind)
  for (i in 1:ngroups){
    cond=uni>res3[,i] & uni<res3[,i+1]
    z[cond]=i
  }
  list(z=z,pi.h=pi.h)
}
#-----------------------------------
update.vh=function(paramz,ngroups,alpha,nind){
  ztab=rep(0,ngroups)
  tmp=table(paramz$z)
  ztab[as.numeric(names(tmp))]=tmp
  n.maior.h=nind-cumsum(ztab)
  tmp1=rbeta(ngroups-1,ztab[-ngroups]+1,alpha+n.maior.h[-ngroups])
  c(tmp1,1)
}
#-----------------------------------
update.psi=function(paramz,ngroups,nloc,epsilon){
  res=matrix(NA,ngroups,nloc)
  for (j in 1:ngroups){
    cond=paramz$z==j
    tmp=dat[cond,]
    if (sum(cond)==0) tmp1=rep(0,nloc)
```

```r
    if (sum(cond)==1) tmp1=tmp
    if (sum(cond)>1)  tmp1=apply(tmp,2,sum)
    res[j,]=rdirichlet(1,tmp1+epsilon)
  }
  res
}
#------------------------------
calc.loglikel=function(paramz,ngroups,nloc){
  loglikel=0
  for (i in 1:ngroups){
    cond=paramz$z==i
    if (sum(cond)>0){
      tmp=sum(dat[cond,]*matrix(log(paramz$psi[i,]),sum(cond),nloc,byrow=T))
      loglikel=loglikel+tmp
    }
  }
  loglikel
}
```

Here is the code within the file "gibbs_functions_cpp.cpp":

```cpp
#include <Rcpp.h>
using namespace Rcpp;

// aaaaaaaaaaaaaaaaaaaaaaaaaaaaaaaaaaaaaaaaaaaa

//dat1z is a matrix of nrows (rows) vs ncols (columns)

//This function calculates the maximum of each line

// [[Rcpp::export]]

NumericVector getmax(NumericMatrix dat1z, int nrows, int ncols) {
  NumericVector res(nrows);
  double max1;

  for (int oo = 0; oo < nrows; oo++){
    max1=-std::numeric_limits<double>::infinity();
    for (int jj = 0; jj < ncols; jj++){
      if (dat1z(oo,jj)>max1) {
        res(oo)=dat1z(oo,jj);
        max1=dat1z(oo,jj);
      }
    }
  }

  return res;
}

// aaaaaaaaaaaaaaaaaaaaaaaaaaaaaaaaaaaaaaaaaaaa

//dat1z is a matrix of nrows (rows) vs ncols (columns)
```

```
//This function calculates cumsum over the rows of dat1z

// [[Rcpp::export]]

NumericVector cumsummat(NumericMatrix dat1z, int nrows, int ncols) {
  NumericMatrix res(nrows,ncols);

  for (int oo = 0; oo < nrows; oo++){
    res(oo,0)=dat1z(oo,0);
    double max1=dat1z(oo,0);
    for (int jj = 1; jj < ncols; jj++){
        max1=max1+dat1z(oo,jj);
        res(oo,jj)=max1;
    }
  }

  return res;
}
```

Benchmarking IBC against current network analysis methods

Simulated data

We created multiple sets of simulated data to compare the performance of our method to that of alternative algorithms. In our first set of simulations, we varied the proportion of mixed membership sites (from 0% to 80%) while assuming the existence of 5 underlying communities. In our second set of simulations, we varied the underlying number of communities from 3 to 10 while setting the proportion of mixed membership sites to 40%. We performed these sets of simulations assuming 40 (small network) and 400 (large network) locations to explore how network size influenced our results.

Alternative algorithms

We compared our IBC method to three existing network analysis methods. The first two methods were hard-clustering methods that have been widely adopted in the literature: the fast modularity optimization algorithm (FMM) proposed by Blondel et al. [1] and the flow-based "Map equation" method (ME) [2]. Both of these methods were implemented using the "igraph" package in R. The third algorithm is a newer method called linked community algorithm (Link) which has generated considerable interest in the scientific community because, similar to our method, it can handle overlapping communities [3]. This method is implemented by the R package "linkcomm".

Differently from the main text, we did not include the modularity optimization approach using the simulated annealing algorithm because it was too slow for the large network. We also tried to use the model-based clustering model of Handcock et al [4] but the algorithm was not converging well despite running very long chains.

Comparison of models

There are multiple ways in which the different clustering algorithms can be compared. Here we compare these algorithm using three criteria. The first one consisted in assessing if these algorithms correctly detected the true number of communities. The second criterion consisted of comparing the link community algorithm and the IBC method (the only two algorithms that allow for mixed membership sites) in relation to their ability to correctly discriminate between mixed membership sites and non-mixed membership sites. This is an important task given the role of these critical connectivity sites in multiple disciplines. For this criterion, we calculate the proportion of sites correctly classified. This is given by $\frac{CM+CNM}{n}$ where n is the total number of sites, and CM and CNM are the number of sites correctly classified as mixed and non-mixed membership sites, respectively.

Our third criterion was based on model fit (as measured by the likelihood). In this approach, we assume that each observation $\boldsymbol{x_i}$ consists on the number of movements originating from location i (i.e., the i-th row of the location-by-location **X** matrix). The total number of movements from this location is given by $n_i = \sum_{j=1}^{J} x_{ij}$. Based on these definitions, we assume that the likelihood is given by:

$$L(\boldsymbol{X}|\boldsymbol{p}) \propto \prod_{i=1}^{I} Multin(\boldsymbol{x_i}|n_i, \boldsymbol{p_i})$$

The hard clustering network analysis approaches we adopted in the manuscript do not explicitly define $\boldsymbol{p_i}$. Nevertheless, we can estimate $\boldsymbol{p_i}$ as follows. Define the probability of movement within group k as $p_w^k = \frac{\sum_{i \in S_k} \sum_{j \in S_k} x_{ij}}{\sum_{i \in S_k} \sum_j x_{ij}}$, where S_k is the set of locations that belong to group k. Using this probability, we define $p_{ij} = \frac{p_w^k}{N_k}$ if locations i and j belong to group k, where N_k is the number of locations that belong to group k. Similarly, we define $p_{ij} = \frac{1-p_w^k}{N_{.}-N_k}$ if location i belongs to group k but location j does not, where $N_{.} = \sum_{k=1}^{K} N_k$ is the total number of locations.

For the IBC method, we assume that p_{ij} is given by:

$$p_{ij} = p(destinity = site\ j|origin = site\ i)$$

$$\propto \sum_{k=1}^{K} p(destinity = site\ j|z = k)p(origin = site\ i|z = k)p(z = k)$$

$$\propto \sum_{k=1}^{K} \psi_{kj}\psi_{ki}\beta_k$$

where z is a variable that denotes the membership status of a randomly chosen individual. Unfortunately, we did not find a simple way to define p_{ij} for the linked community algorithm and therefore we do not use the likelihood criterion to compare this method to the other algorithms.

Results

Our IBC method generally tended to correctly estimate the number of communities (Fig. A1). However, there were some discrepancies, particularly when the number of communities was large (≥ 7) and when a very high proportion of sites (≥ 60%) were mixed for the large network. The hard-clustering methods tended to almost always underestimate the true number of communities. In particular, the map equation algorithm surprisingly estimated that just a single community was present in most of our simulations. This result illustrates how having even a modest amount of mixed membership sites can substantially hinder the ability of some of these network analysis algorithms to identify the underlying patterns in these data. Finally, despite the ability to represent mixed sites, the link community algorithm failed to correctly identify the true number of communities.

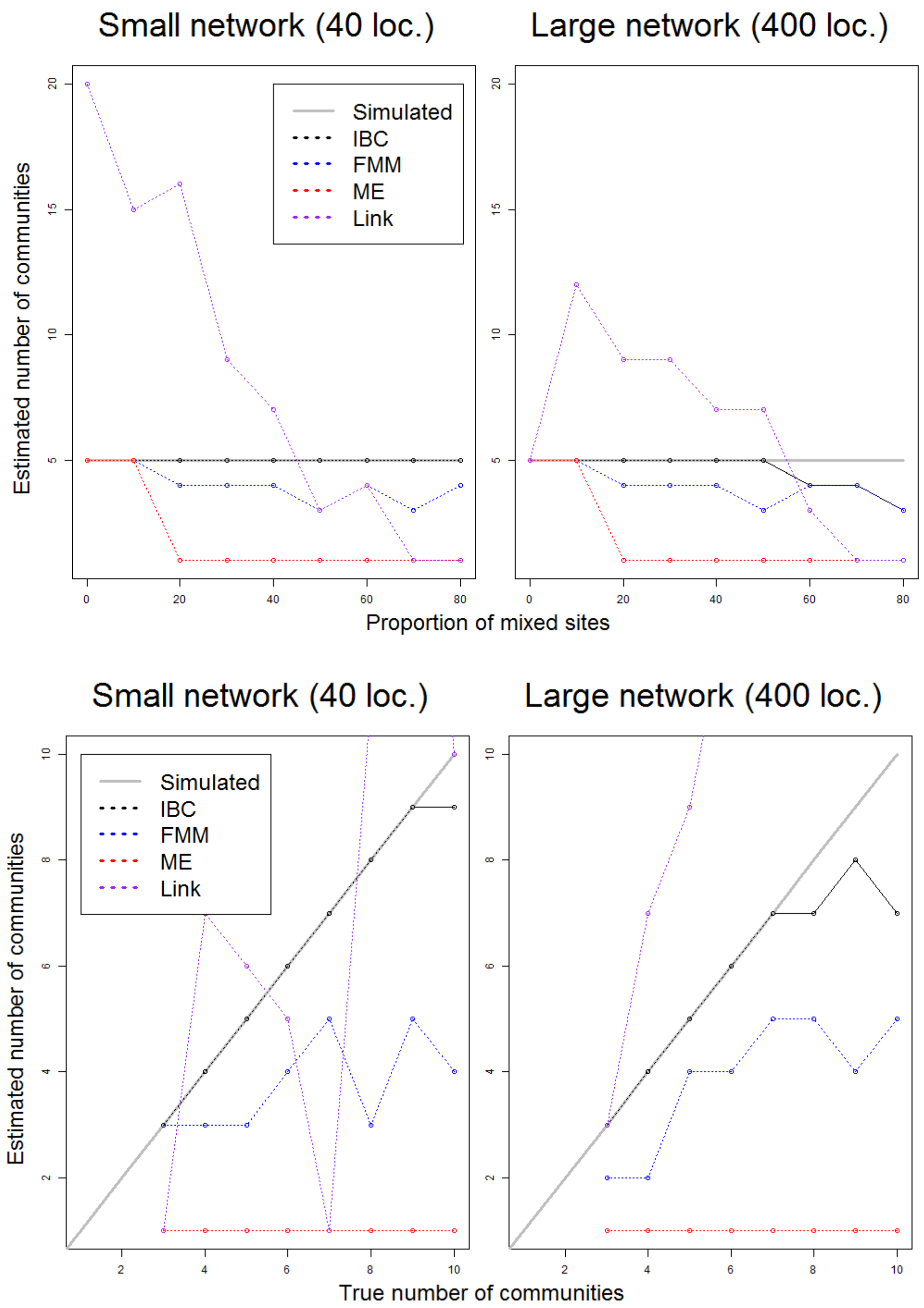

Fig. A1. Comparison between the estimated (y-axis) and the true number of communities. Top panels depict the results for our first set of simulations, in which the true number of communities was set to 5 (grey horizontal line) and the proportion of mixed sites varied from 0 to 80% (x-axis). Bottom panels depict the results for our second set of simulations, in which the true number of communities varied from 3 to 10 (x-axis) and the proportion of mixed sites was set to 40%. Diagonal grey line is a 1:1 line in the bottom panels.

We also assessed how well the link community algorithm and the IBC method were able to detect mixed membership sites. While the IBC method almost always correctly identified mixed

membership and non-mixed membership sites, the link community algorithm had a substantially worse performance (Fig. A2). Finally, in relation to model fit, our results reveal that IBC had a substantially better fit than the other two hard-clustering methods (Fig. A3).

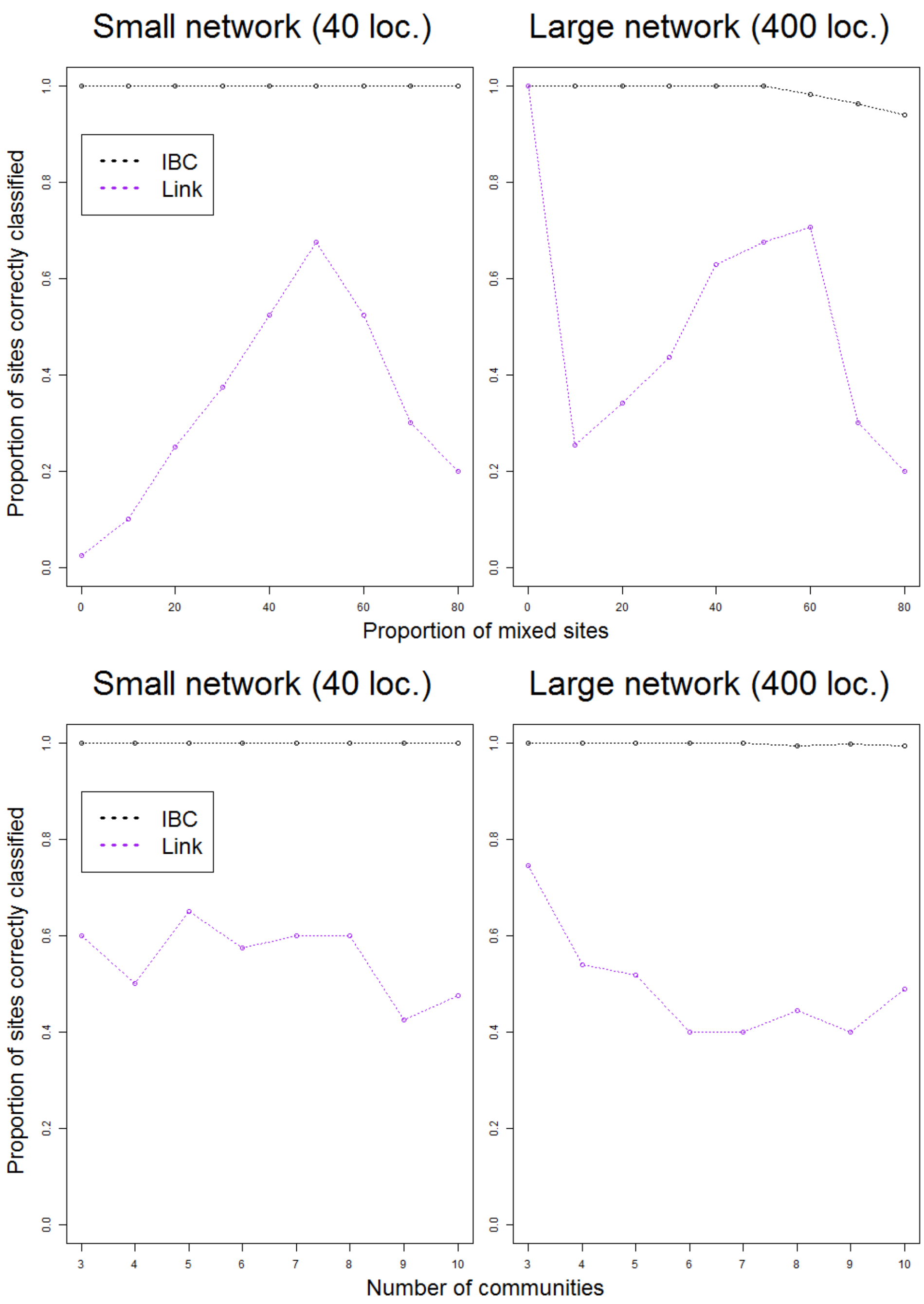

Fig. A2. Comparison of IBC and the Link algorithm in predicting mixed and non-mixed membership sites. Values closer to 1 indicated better fit to the data. Top panels depict the results for our first set of

simulations, in which the true number of communities was set to 5 and the proportion of mixed sites varied from 0 to 80% (x-axis). Bottom panels depict the results for our second set of simulations, in which the true number of communities varied from 3 to 10 (x-axis) and the proportion of mixed sites was set to 40%.

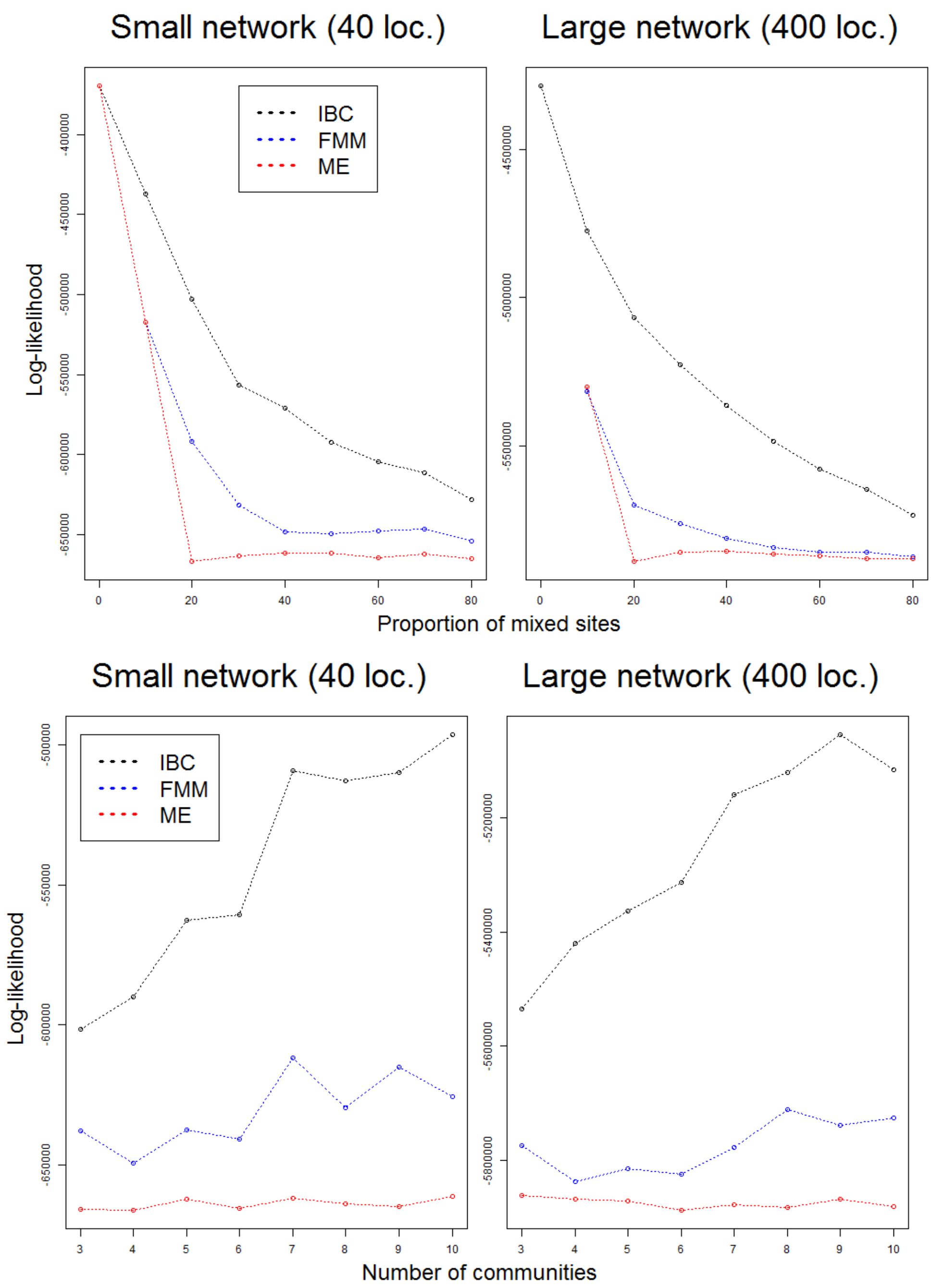

Fig. A3. Comparison of model fit (as assessed by the log-likelihood, y-axis) for different algorithms. Higher values indicated better fit to the data. Top panels depict the results for our first set of simulations, in which the true number of communities was set to 5 and the proportion of mixed sites varied from 0 to 80% (x-axis). Bottom panels depict the results for our second set of simulations, in

which the true number of communities varied from 3 to 10 (x-axis) and the proportion of mixed sites was set to 40%.

References

1 Blondel, V. D., Guillaume, J.-L., Lambiotte, R. & Lefebvre, E. Fast unfolding of communities in large networks. *J Stat Mech Theor Exp* **P10008** (2008).

2 Rosvall, M. & Bergstrom, C. T. Maps of random walks on complex networks reveal community structure. *Proc Natl Acad Sci USA* **105**, 1118-1123 (2008).

3 Ahn, Y.-Y., Bagrow, J. P. & Lehmann, S. Link communities reveal multiscale complexity in networks. *Nature* **466**, 761-764 (2010).

4 Handcock, M. S., Raftery, A. E. & Tantrum, J. M. Model-based clustering for social networks. *J Roy Stat Soc A Sta* **170**, 301-354 (2007).
